# In silico decrypting of the bystander effect in antibody–drug conjugates for breast cancer therapy

**DOI:** 10.1038/s41598-025-13810-w

**Published:** 2025-08-06

**Authors:** Víctor L. Cruz, Jorge Duro-Sánchez, Emma Franco-Mateos, Laura García-Estévez, José Pérez-García, María Gion, Laia Garrigós, Patricia Cortez, Cristina Saavedra, Patricia Gómez, Carolina Ortiz, Arantxa Aguinagalde, Javier Ramos, Javier Cortés, Juan F. Vega

**Affiliations:** 1 BIOPHYM, Departamento de Física Macromolecular, Instituto de Estructura de la Materia, IEM-CSIC, Madrid, Spain; 2 Breast Cancer Department, MD Anderson Cancer Center, Madrid, Spain; 3 International Breast Cancer Center (IBCC), Pangea Oncology, Quironsalud Group, Barcelona, Spain; 4 Medica Scientia Innovation Research (MEDSIR) – Oncoclínicas & Co, Jersey City, NJ USA; 5 Medica Scientia Innovation Research (MEDSIR) – Oncoclínicas & Co, Sao Paulo, Brazil; 6 Medica Scientia Innovation Research (MEDSIR) – Oncoclínicas & Co, Barcelona, Spain; 7 Medical Oncology Department, Ramón y Cajal University Hospital, Madrid, Spain; 8 IOB Madrid, Institute of Oncology, Beata Maria Ana Hospital, Madrid, Spain; 9 Faculty of Biomedical and Health Sciences, Department of Medicine, Universidad Europea de Madrid, Madrid, Spain

**Keywords:** Antibody–drug conjugates, Bystander effect, Molecular dynamics simulations, Payload ionization and membrane permeability, Linker design, Cancer, Computational biology and bioinformatics, Drug discovery, Oncology

## Abstract

Antibody–Drug Conjugates (ADCs) are a promising cancer treatment that deliver toxic drugs directly to cancer cells, reducing harm to healthy tissue. A key feature of newer ADCs is the “bystander effect,” in which nearby cancer cells are also affected by passive diffusion. However, the mechanisms underlying this effect remain unclear. Using computer simulations, this study investigates how the drug’s ionization state and the linker connecting it to the antibody influence its ability to cross cell membranes. The results show that the ionization state of the drug impacts its membrane permeability, as charged molecules encounter resistance when moving through the membrane’s hydrophobic core. Moreover, the study reveals that the linker increases the drug’s overall size and hydrophobicity, thereby hindering its diffusion to adjacent cells. This finding suggests that linker design can significantly influence the efficacy of antibody–drug conjugates (ADCs) by limiting their ability to reach neighboring cancer cells. These insights enhance our understanding of ADC mechanisms and provide a valuable foundation for the optimization of next-generation ADC therapies targeting breast cancer.

## Introduction

Antibody–Drug Conjugates (ADCs) stand at the forefront of targeted cancer treatment, holding promise as a replacement for conventional chemotherapies^1^. ADCs are sophisticated agents involving the binding of antibodies to specific target antigens on tumor cells. The classical mechanism of action starts with the internalization of the ADCs, followed by linker breakdown and the release of cytotoxic payloads within tumor cells, inducing cell death. However, recent studies indicate that, depending on the nature of the linker and the payload, the internalization of ADCs is not the only way to exert the activity. ADCs can be cleaved extracellularly, releasing the payload, which can then enter the cellular cytosol via passive diffusion^2^. Intriguingly, certain ADCs demonstrate a *bystander effect*, wherein the released cytotoxic agent can eradicate adjacent tumor cells that do not express the target antigen by passive diffusion through the cellular membrane^3–6^. Although this property is not yet fully understood, factors like the chemical features of the payload and the rest of linker moieties may influence the bystander effect and overall efficacy of the ADC^2,7,8^.

While the bystander effect holds great potential for enhancing therapeutic results, experimental studies to investigate this phenomenon face challenges^9^. Among these, a significant difficulty lies in achieving optimal spatial and temporal resolution of the bystander mechanism, particularly in relation to the diffusion of the payload across the cellular membrane^10,11^. Traditional experimental techniques often lack the necessary resolution to accurately capture transient and localized events such as payload diffusion and distribution after internalization. In fact, observing interactions between targeted and neighboring cells at the molecular level in real-time remains technically difficult^12^.

In silico approaches offer valuable solutions to address the challenges of studying passive diffusion. In particular, homology modelling, molecular docking, and atomistic and coarse-grained molecular dynamics (MD) simulations may be applied for a full characterization of the structure—activity relationship of ADCs for research and development^13^. MD simulations provide precise control over chemical, structural, and topological parameters of ADCs, enabling atomistic-scale insights into their interactions with cellular components, including the cell membrane, as well as their dynamics. Furthermore, these tools present a cost-effective and time-efficient alternative to expensive experimental studies. Despite the wealth of theoretical models in the literature, employing non-equilibrium dynamics^14,15^, metadynamics^16^, quantum-based models^17^ or chemometric tools^18^, there is a gap in the in silico exploration of the specific payloads/linkers integral to ADC therapies. This study aims to fill this gap by providing new insights into payloads and linker behavior through in silico simulations. It should be noted that passive diffusion of the payload and/or linker through the membrane lipid bilayer may represent a simple mechanism to study the bystander effect. Other possible mechanisms, not studied here, include extracellular cleavage followed by diffusion in the tumor interstitium, active efflux pumps, and transfer via extracellular vesicles. However, passive diffusion may be more amenable to theoretical atomistic analysis, potentially providing sufficient detail to differentiate compounds with and without a bystander effect.

Our attention centers on three important cytotoxic payloads—deruxtecan, auristatin, and maytansinoid—widely used for breast cancer treatment. The prevalent deruxtecan variants, specifically S-8201a (DXd1) and DXd2, are integral components of ADCs due to their derivation from exatecan. Deruxtecan, recognized as a potent topoisomerase I inhibitor, stands out for its ability to induce DNA damage within cancer cells, demonstrating pronounced efficacy, particularly against HER2-positive breast cancer, a formidable tumor subtype^19^. Auristatin payloads, comprising monomethyl auristatin E and F (MMAE and MMAF), belong to a potent class of microtubule-disrupting agents, effectively arresting cell division and triggering apoptosis in cancer cells^20^. Maytansinoid payloads form part of ado-trastuzumab emtansine, the first ADC approved for the treatment of solid tumors^3^. Clinical trials featuring ADCs with these payloads demonstrate significant tumor regression and improved patient outcomes. Current investigations also explore combinations of these ADCs with other drugs, highlighting their potential synergy^21^. We chose these closely related payload groups due to experimental evidence of the bystander effect in one member of each group. That is the case for DXd1 and MMAE, demonstrating bystander effect. In the case of emtansine, the linker is not cleaved from the maytansinoid, resulting in the release of a molecule containing a zwitterion species in physiological pH conditions, which is supposed to inhibit diffusion through a membrane and, consequently lacks bystander effect^22^.

This work investigates the behavior of these payloads using well-established in silico models at atomic resolution. Despite significant advancements in ADC development, the mechanistic understanding of the bystander effect remains incomplete. Previous research has often focused on either clinical observations or limited computational models. More recently, computational methods based on artificial intelligence, specifically utilizing Graph Attention Networks (GATs), a deep learning approach designed for processing graph-structured data, has been used to model and optimize the bystander effect in ADC^23^. More than this, the study provides the first comprehensive in silico atomic-level analysis of how both payload ionization and linker design influence the bystander effect in ADCs. The atomic-level simulations reveal the specific structural and energetic factors that modulate membrane permeability and, consequently, bystander killing. These insights offer a new framework for rational ADC design, aiming to enhance therapeutic outcomes in breast cancer.

## Results

We aim to elucidate how both the ionization state of payloads and the presence of linkers influence the bystander effect in ADCs. Specifically, we investigate whether payloads with different charge states and linker sizes are capable of passively diffusing through a lipid bilayer—an essential step for bystander killing. Additionally, other parameters were evaluated for their potential influence on the likelihood of a bystander effect, including hydrogen-bonding capacity, free energy barriers (such as flip-flop and desorption), and the molecular size of different payloads, as measured by their radius of gyration (R_g_).

**Fig. 1:**
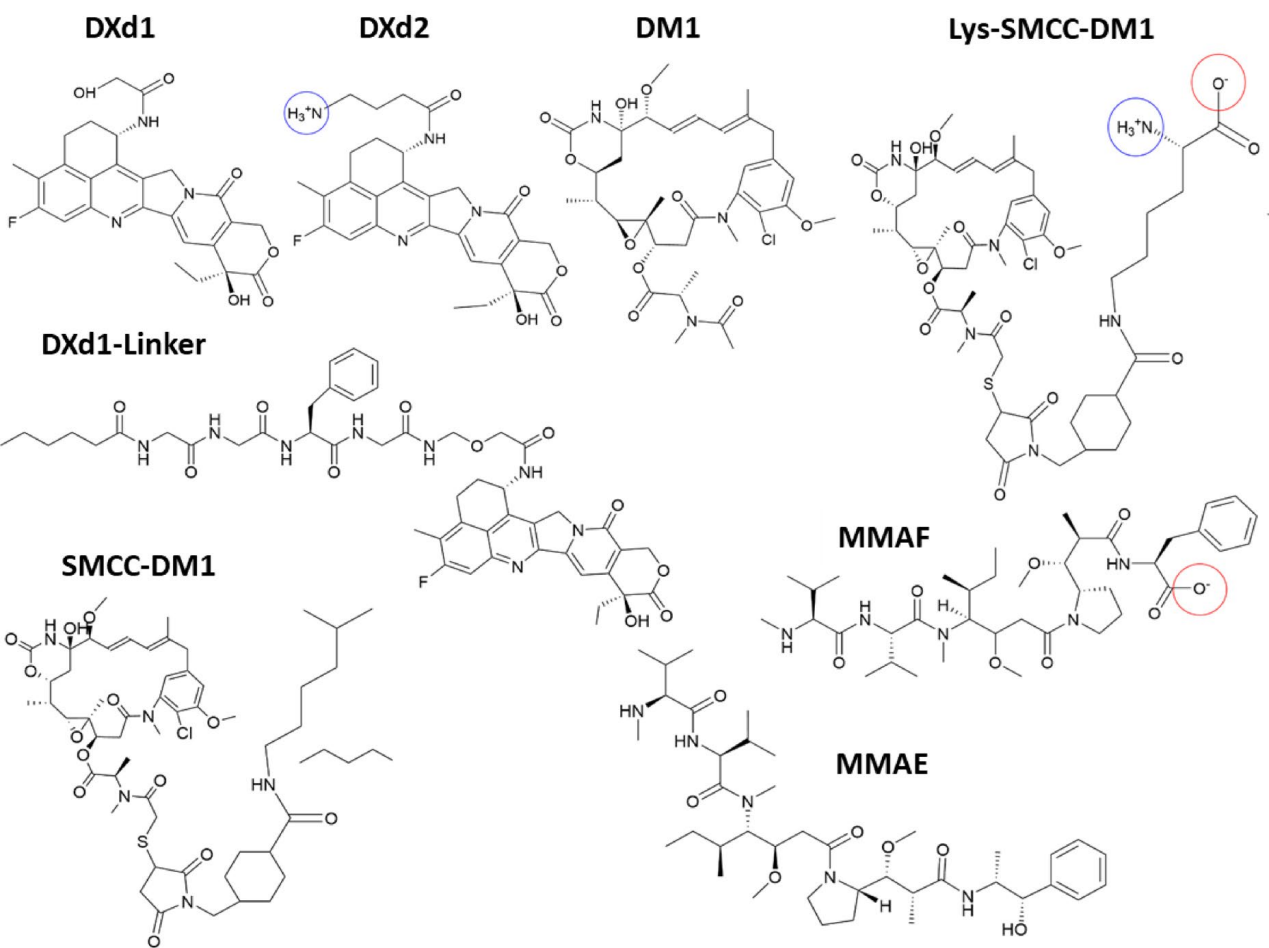
Chemical structure of the payloads studied. Ionizable groups are highlighted (circles). Payloads lacking linker moieties include deruxtecan (DXd1 and DXd2) and auristatins (MMAE and MMAF). The emtansine payload incorporates a non-cleavable linker (Lys-SMCC-DM1). The maytansinoid base molecule (DM1) is compared with the reference Lys-SMCC-DM1 payload. DXd1-linker and SMCC-DM1 are specifically analyzed to assess the role of the linker in the bystander effect (see main text for details).

Some of the payloads examined in this study (Fig. 1) contain ionizable groups that become charged at physiological pH values between 5 and 7. To determine the ionization state of the payloads under these conditions, their corresponding pKa values were evaluated, as detailed in the Methods section. Specifically, DXd2 features an alkyl-amino group that becomes protonated to form –NH_3_^+^, while MMAF contains a carboxylate group that remains deprotonated at these pH levels. Additionally, the Lys-SMCC-DM1 combination of an uncleavable linker and payload may result in a zwitterionic species after lysosomal ADC digestion. Throughout the remainder of this description, these molecules are considered in their ionized states as determined by the pKa analysis.

### Evaluation of the pKa

The following pKa values were obtained for the ionizable molecules: DXd2, 10.4 ± 2.4; and MMAF, 2.2 ± 0.9. Lys-SMCC-DM1 has two ionizable groups with calculated pKa (COO^−^) = 2.2 ± 0.9 and pKa (NH_3_^+^) = 9.0 ± 0.9. These results are indicative that, at physiological pH, DXd2 is protonated, MMAF is deprotonated, and Lys-SMCC-DM1 is zwitterionic.

### Molecular dynamics simulation results

Before presenting the simulation data, it is important to note that understanding passive diffusion of payloads across a lipid bilayer requires assessing both flip-flop across the membrane and desorption from the membrane–water interface. Here, the initial steps taken to prepare the systems are described, including pKa determination, force field selection, and the assembly of POPC bilayers with solvated payloads. Extensive MD simulations were performed to capture steady-state configurations, followed by potential of mean force (PMF) calculations to quantify the free energy barriers for crossing (flip-flop) and leaving (desorption) the bilayer.

**Fig. 2:**
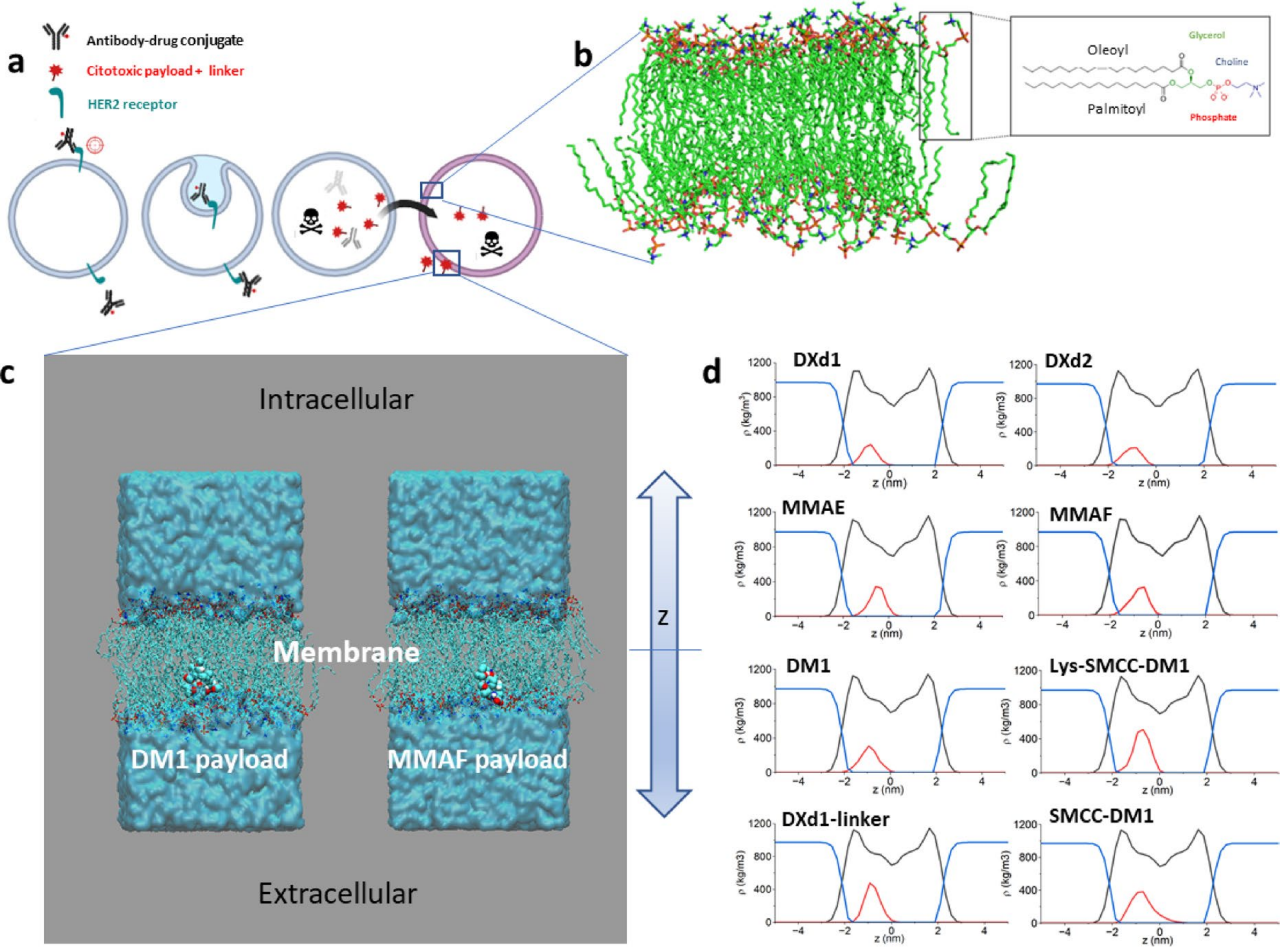
The bystander effect and the in silico  approach in this study. (**a**) Schematic representation of the bystander effect. (**b**) Detail of a model of lipid bilayer: The lipid bilayer structure used in all cases corresponds to 64 Palmitoyl Oleoyl Phosphatidyl Choline (POPC). Image created using VMD software version 1.9.4. (**c**) Final snapshot of the production MD simulation for DM1 and MMAF payloads. Payload molecules are represented by VDW spheres, colored by atom type. POPC lipids are depicted as lines with the same coloring criterion and water is shown as a cyan surface representation. (**d**) Mass density profiles for the three payload systems. Profiles for the payload molecules were multiplied by ten to allow a better visualization: POPC in black, payload in red, and water in blue.

Figure 2a schematically illustrates the passive diffusion process targeted in the simulations. After the internalization of the ADCs and the release of the cytotoxic drug within the tumor cells the bystander effect may proceed by passive diffusion of the payload across the lipid bilayer (Fig. 2b). The lipid bilayer structure used in all cases is composed of 64 Palmitoyl-Oleoyl-Phosphatidylcholine (POPC) (see Methods section). In all cases, unrestricted MD simulations resulted in the adsorption of the payload at the interface between the lipid polar head groups and the hydrocarbon core of the lower (extracellular) leaflet of the bilayer.

Figure 2c illustrates the cases of MMAE and MMAF as representative examples of positive and negative bystander effects, respectively. All the molecules exhibit a similar profile relative to the lipid layer, consistent with the visual representation shown in the figure. The averaged mass density profiles along the bilayer normal of all systems, as depicted in Fig. 2d, provide insight into the relative positioning of payloads within the bilayer interior across the simulations. These payload molecules are amphiphilic compounds that partition comfortably in the amphiphilic interface of one of the lipid leaflets. All the payloads contain hydrogen-bond donors capable of forming hydrogen bonds with an oxygen atom acceptor in the lipid molecules, namely, the two non-bridging oxygen atoms in the phosphate group and the two carbonyl oxygen atoms of the acyl chains.

In Table 1, the number of available hydrogen bond donors in each payload is listed along with the number of hydrogen bonds averaged over the production trajectory. The number of hydrogen bonds were calculated using the following criterion: the distance donor–acceptor atoms is less than 0.35 nm and the angle between hydrogen donor–acceptor is below 30°. This number is higher in the protonated molecules DXd2 and Lys-SMCC-DM1 than for the unprotonated DXd1 and DM1, respectively, as anticipated due to the additional hydrogen atom in the amine group and its geometric proximity to the negatively charged lipid phosphate group.

**Table 1:** Properties of the cytotoxic payloads studied.

Payload	Potential H-bond donors^a^	Averaged H-bonds^b^	ΔG flip-flop^c^ (kJ mol^−1^)	ΔG desorption^d^ (kJ mol^−1^)	Bystander effect	AlogP^e^	R_g_^f^ (nm)
DXd1	3	1.3	20 ± 5	40 ± 5	Yes	− 0.25	0.47 ± 0.02
DXd2	5	2.3	80 ± 5	40 ± 5	No	− 0.15	0.49 ± 0.02
MMAE	4	0.7	15 ± 5	50 ± 5	Yes	3.10	0.55 ± 0.02
MMAF	3	0.6	75 ± 5	50 ± 5	No	2.70	0.56 ± 0.02
DM1	2	1.3	20 ± 5	55 ± 5	Yes	3.00	0.47 ± 0.02
Lys-SMCC-DM1	6	2.3	65 ± 5	75 ± 5	No	4.00	0.62 ± 0.02
DXd1-Linker	7	2.3	20 ± 5	80 ± 5	No	1.05	0.75 ± 0.02
SMCC-DM1	3	1.1	25 ± 5	75 ± 5	No	7.10	0.70 ± 0.02

^a^Number of potential hydrogen bond donors in each payload (based on the functional groups able to donate H-bonds). ^b^Average number of hydrogen bonds formed between the payload and lipids during the last 1 µs of the MD simulation (distance < 0.35 nm and angle < 30°). ^c^Free energy barrier (in kJ mol⁻^1^) for the payload to traverse the hydrophobic interior of the lipid bilayer (flip-flop). ^d^Free energy barrier (in kJ mol⁻^1^) for the payload to desorb from the lipid–water interface into the aqueous phase. ^e^AlogP: Partition coefficient calculated via the ALogP model, indicating relative hydrophobicity. ^f^Radius of gyration (in nm), reflecting the average molecular size of the payload over the MD trajectory.

**Fig. 3:**
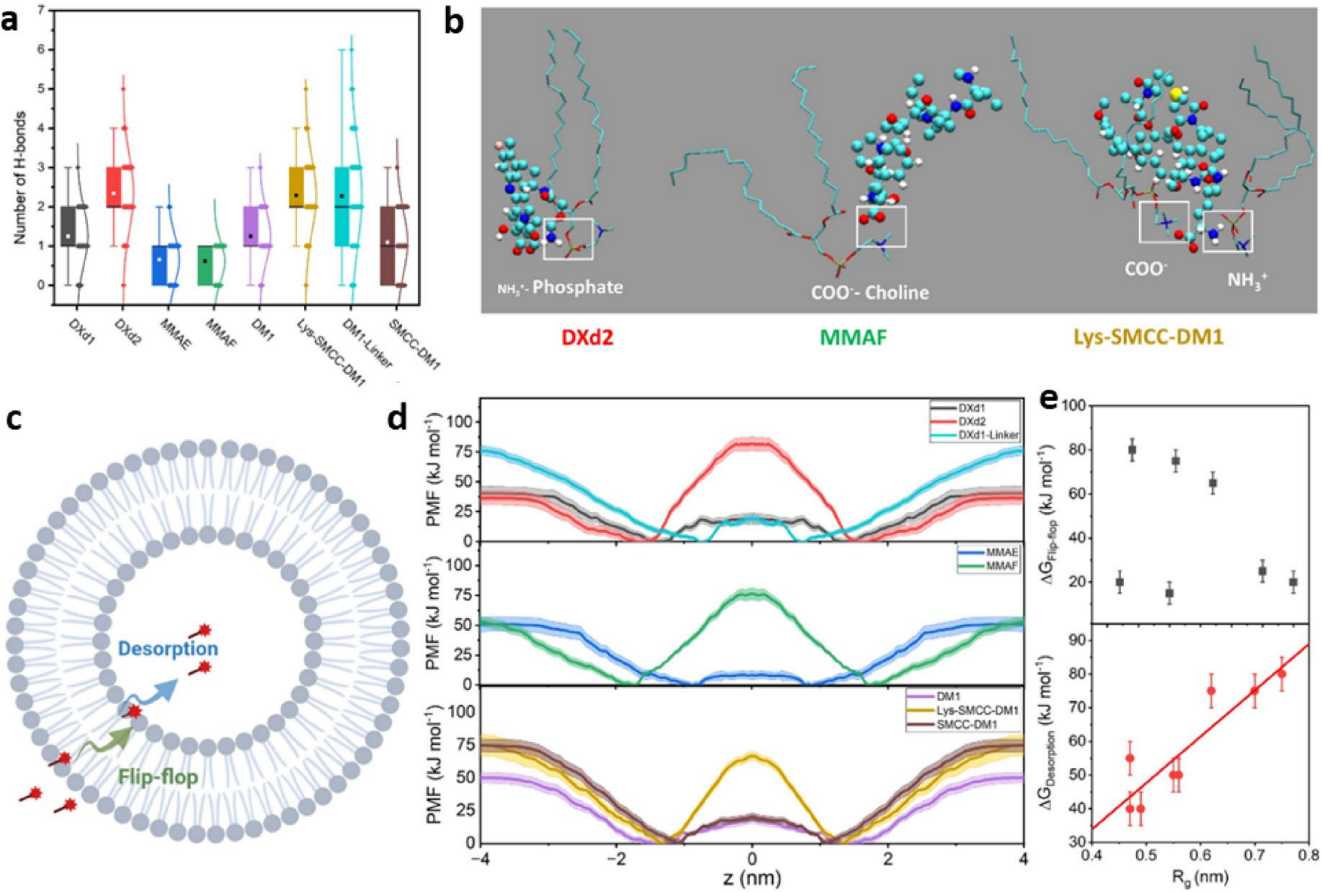
Effect of H-bonding and electrostatic interactions in bystander effect. (**a**) Number of hydrogen bonds along the MD simulations. (**b**) Molecular structures illustrating the electrostatic interactions between charged payloads and POPC molecules. The payloads are depicted in ball-stick representation colored by atom type, and the POPC as sticks with the same pattern scheme. Image created using VMD software version 1.9.4. (**c**) Schematic representation of the two steps for the payload permeation by passive diffusion through the lipid bilayer. (**d**) PMF profiles for the payloads’ permeation of POPC. Top: Deruxtecan derivatives. Center: Auristatin derivatives. Bottom: Maytansinoid derivatives. (**e**) Correlation between the size of the linker, expressed as radius of gyration (R_g_), and the energy barriers (ΔG) of the two stages during the passive diffusion process.

The box plot Fig. 3a shows the statistics of hydrogen bonds occurrence along the simulation time for each of the payload studied. An important difference between the payloads with and without bystander effect is the presence of charged groups in the latter case. This feature involves the occurrence of electrostatic interactions between those charged groups and the positively charged choline and/or negatively charged phosphate groups of the lipid molecules. Figure 3b illustrates these interactions, extracted from various MD snapshots. The positively charged NH_3_^+^ group consistently resides at distances below 0.5 nm from the POPC phosphate groups. Additionally, the amine hydrogens participate in hydrogen bond with the carbonyl oxygen of the lipid tails, as depicted in Table 1 and Fig. 3b. In the case of the MMAF negatively charged carboxylate group, a persistent interaction with the positively charged choline group is observed. Finally, a combination of these two interactions is present in the case of the zwitterionic Lys-SMCC-DM1 payload.

### Potential of mean force results

The potential of mean force (PMF) value was computed for two key steps in the diffusion process, namely, payload “flip-flop” across the bilayer interior and desorption from the lipid bilayer, as described in the Methods section. Figure 3c illustrates these two steps within the passive diffusion process. Initially, the payload crosses through the hydrophobic bilayer interior. Subsequently, upon completing this phase, the payload desorbs from the lipid polar/apolar interface, ultimately reaching the aqueous phase on the other side of the membrane. The reader is referred to the Supplementary Video, in which the process of the diffusion of the DXd1 (left) and DXd2 (right) payloads (with and without bystander effect, respectively) across the lipid bilayer is observed.

The PMF simulations for both the flip-flop and desorption steps started for the initial structure obtained from unrestricted MD simulations (Fig. 2c). Figure 3d depicts the combined PMFs for each payload molecule, illustrating their perpendicular movement (in opposite directions) relative to the lipid bilayer surface during the flip-flop and desorption processes. Table 1 serves a complementary resource, providing the calculated ΔG energy barriers for these steps. These data provide valuable insights into the energetic demands associated with the transition of each payload molecule within the lipid bilayer. The three payload groups share a common observation: the ionized molecules present large barriers crossing the hydrophobic bilayer phase (80 kJ mol^−1^ for DXd2, 75 kJ mol^−1^ MMAF, and 65 kJ mol^-1^ for Lys-SMCC-DM1). This circumstance, typical in drug permeability through membranes, allows us to explain the lack of bystander effect behavior of those payloads, as they remain retained on one leaflet of the lipid bilayer. In contrast, electrostatically neutral payloads such as DXd1, MMAE, and DM1, which have shown a bystander effect^24–27^, present much lower energy barriers (20 kJ mol^−1^ for DXd1, 15 kJ mol^−1^ for MMAE and 20 kJ mol^−1^ for DM1compared to the ionized compounds). The desorption step to enter the water phase presents free energy barriers in the range 40–55 kJ mol^−1^ for linker-cleaved payloads. The zwitterionic Lys-SMCC-DM1 presents a larger barrier of around 75 kJ mol^−1^. Conversely, the uncleaved linkers DXd1-Linker and SMCC-DM1 show a relatively small flip-flop energy barrier (20 kJ mol^−1^ and 25 kJ mol^−1^ for the DXd1-Linker and SMCC-DM1 molecules, respectively).

The desorption energies are higher than the other molecules considered (80 kJ mol^−1^ for DXd1-Linker and 75 kJ mol^−1^ for SMCC-DM1). The similarity observed between the Lys-SMCC-DM1, DXd1 linker, and SMCC-DM1 suggests that the linker contributes to the effect beyond merely the presence of ionizable groups. Hydrophobicity and molecular size are recognized as factors that influence the molecular permeability^15,28^. Hydrophobicity can be estimated using the AlogP fragmental model, which is based on empirical data from a large set of molecules^29^. The radius of gyration (R_g_), averaged over the production MD simulation, provides an estimate of the molecular size. Table 1 summarizes the values for AlogP and R_g_ for the different payloads. Interestingly, payload size appears to negatively impact the desorption process. Larger values, corresponding to the uncleaved linker payloads (Lys-SMCC-DM1, SMCC-DM1 and DXd1-Linker), correlate with the higher ΔG values for the desorption process, as observed in Table 1 and Fig. 3e. However, the influence of hydrophobicity, as calculated with the AlogP model, seems to be marginal for both the flip-flop and the desorption steps. For instance, despite different AlogP values for DXd1 and MMAE (− 0.25 and 3.10, respectively; see Table 1) their flip-flop ΔG values are comparable (20 kJ mol^−1^ and 15 kJ mol^−1^, respectively; see Table 1). A similar observation can be made when comparing the AlogP values of DM1, SMCC-DM1 and DXd1-Linker molecules (3.0, 7.1 and 1.05, respectively; see Table 1) with their flip-flop ΔG values (20 kJ mol^−1^, 25 kJ mol^−1^ and 20 kJ mol^−1^, respectively; see Table 1).

## Discussion

ADCs have emerged as some of the most active drugs in cancer treatment^1^. This study demonstrates the critical influence of payload ionization and linker characteristics on ADC design, particularly regarding their effects on membrane permeability and the bystander effect. The results highlight the importance of understanding the fundamental mechanisms of passive diffusion, including the flip-flop and desorption processes, to optimize the therapeutic efficacy of ADCs.

### Mechanisms of passive diffusion

Passive diffusion across cell membranes consists of two primary processes: flip-flop and desorption. The flip-flop mechanism involves the movement of molecules from the aqueous phase into the hydrophobic core of the lipid bilayer and vice versa. This process is pivotal for the transport of payloads into cells, as the energy required to traverse the membrane depends on the molecular nature of the payload. Neutral payloads, such as DXd1 and MMAE, exhibit relatively low flip-flop energy barriers (approximately 15–20 kJ mol^−1^), which facilitates their movement across the membrane, resulting in a positive bystander effect. In contrast, payloads with ionizable groups, such as DXd2 and MMAF, show significantly higher flip-flop energy barriers (around 75–80 kJ mol^−1^), which impedes their passive diffusion and leads to a negative bystander effect. This discrepancy is attributed to the increased difficulty charged groups face when crossing the lipid bilayer.

Our results specifically indicate that the flip-flop step acts as the rate-limiting factor for all the payloads considered in this study. While desorption also plays a role in releasing the payload from the lipid bilayer, the energy barriers for this process were relatively similar across all cases studied (approximately 40–55 kJ mol^−1^). This observation highlights the predominance of the flip-flop mechanism in determining the bystander effect, suggesting that optimizing this process could enhance ADC efficacy.

### The role of linker chemistry

An intriguing result emerged from the analysis of the uncleavable Lys-SMCC-DM1 payload, which demonstrated similar energy barriers for both the flip-flop and desorption processes, around 70 kJ mol^−1^. This similarity can be attributed to the interplay between the electrostatic effects of ionized groups and the molecular hydrophobicity of the payload. The zwitterionic nature of Lys-SMCC-DM1 facilitates electrostatic and hydrogen bond interactions with lipid molecules, impeding the flip-flop process. At the same time, the increased hydrophobicity of the maytansinoid payload and the linker promotes favorable interactions with lipid hydrocarbon tails, aiding the flip-flop process. As a result, the energetic barrier for Lys-SMCC-DM1 is slightly lower than that observed for ionizable payloads like DXd2 and MMAF (65 kJ mol^−1^versus 80 kJ mol^−1^and 75 kJ mol^−1^, respectively). Furthermore, the linker chemistry appears to influence both the flip-flop and desorption processes. For example, the linker-free maytansinoid DM1, a neutral molecule, exhibits a low desorption energy barrier comparable to that of DXd1 or MMAE, suggesting its potential for a bystander effect. In contrast, neutral uncleaved linkers such as DXd1-Linker and SMCC-DM1 hinder the desorption of the drug from the lipid bilayer interior. This is reflected in desorption energy barrier values of 75–80 kJ mol^−1^, which are associated with the absence of a bystander effect. This observation highlights the negative impact of increased hydrophobicity and linker size on the desorption process, particularly in the maytansinoid Lys-SMCC-DM1, which presents a desorption barrier of 75 kJ mol^−1^.

### Implications of ionization and linker properties

The results indicate that the ionization state of payloads can significantly limit their passive diffusion across the lipid bilayer. The two payloads with ionizable groups—DXd2 and MMAF—are relatively insensitive to physiological pH gradients due to their respective pKa values (DXd2: 10.4 ± 2.4; MMAF: 2.2 ± 0.9). As a result, they remain in their ionized forms, which hinders their escape from the lipid bilayer. Only under extreme pH conditions (pH > 10.4 for DXd2 or pH < 2.2 for MMAF) might the neutral forms become accessible, increasing the likelihood of passive diffusion of the payload through the bilayer. Additionally, linkers with bulky structures or permanent charges have been observed to increase the desorption barrier and hinder bilayer transport. This is in agreement with Giugliani et al. ^7^ and Ogitani et al.^26,27^, who reported that greater structural complexity in the payload reduces the probability of its release into neighboring cells. Taken together, these findings suggest that payload neutrality and linker simplicity favor bilayer permeability, thereby enhancing the bystander effect.

These findings are consistent with previous studies, which highlight how both the chemical properties of the payload and the linker influence drug release, diffusion, and ultimately, the bystander effect^2,7^. At the same time, these results reinforce the hypothesis that modifying the physicochemical properties of both the payload and the linker could be a viable strategy to optimize the efficacy of ADCs in cancer therapies^23,30,31^. A reduction in the energy barriers for both flip-flop and desorption could potentially enhance the therapeutic index of ADCs, facilitating improved delivery of cytotoxic agents to neighbouring cells and reducing off-target effects.

### Computational findings compared with previously reported experimental works

The computational findings align with previously reported experimental data, supporting the validity of the applied approach. The observed bystander effect in DXd1 and MMAE is consistent with experimental studies demonstrating their ability to diffuse across membranes^7,26,27^. While direct experimental studies on DXd2 and MMAF payloads are not available (to the best of our knowledge), several studies have investigated the general impact of molecular charge on membrane permeability. For instance, Liu et al. and Yamaguchi et al. reported that charged molecules display restricted diffusion across zwitterionic membranes, consistent with those utilized in this work^32,33^.

### Broader implications

The insights gained from this study have broad implications for ADC design and optimization. Our findings provide both theoretical and practical knowledge for researchers and clinicians working on targeted cancer therapies. By integrating computational modeling with drug development strategies, this study is highly relevant for oncologists, clinical researchers exploring novel ADC therapies for cancer, and medical chemists focusing on linker chemistry and payload optimization. Furthermore, computational biologists and bioinformaticians involved in in silico drug discovery, as well as professionals in the pharmaceutical industry working on ADC-based treatments, will benefit from these results. Academic institutions studying targeted cancer therapies and membrane transport mechanisms can also leverage these findings to guide future research in ADC development.

Looking ahead, future studies could explore the role of membrane components such as cholesterol, which influences passive diffusion by modulating membrane fluidity and permeability. Increased cholesterol content generally reduces membrane fluidity and enhances hydrophobicity, decreasing permeability to molecules ranging from small species like molecular oxygen to larger nanoparticles^34,35^. However, the effects of cholesterol are complex and may depend on factors such as the specific lipid composition of the membrane and the physicochemical properties of the diffusing molecules. Investigating these aspects will further refine our understanding of ADC transport mechanisms and aid in the design of more effective therapies.

## Methods

### Structural models

#### Structure of anti-HER2 ADC payloads

Three cytotoxic payloads currently used in ADC therapies have been examined, namely, deruxtecan (DXd1 and DXd2)^26^, auristatins (MMAE and MMAF)^36^, and emtansine^3^. The two deruxtecan derivatives differ in the hanging group resulting from the linker cleavage process. The presence of a self-immolative group as part of the DXd1 ADC linker system leads to a primary hydroxyl group, whereas DXd2 retains an amino group coming from the tetrapeptide cleavage step^37^. Auristatins MMAE and MMAF are composed of five amino acids. MMAE consists of norephedrine, dolaproline, dolaisoleuine, valine, and monomethyl valine. In MMAF, the C-terminal norephedrine is replaced by phenylalanine, resulting in a carboxylate end group. For those auristatins, the lowest energy cis conformer, which corresponds to the drug bioactive form, was selected^38,39^. Regarding the emtansine payload, it has been considered the non-cleavable linker as part of the simulated molecule (Lys-SMCC-DM1). In addition to the maytansinoid base molecule (DM1) taken for comparison with the reference Lys-SMCC-DM1 payload^40^, two additional compounds have been studied to assess the role of the linker in the bystander effect. The first compound was constructed by integrating the tetrapeptide linker found in deruxtecan into the DXd1 payload, simulating a scenario where it remains attached to the drug molecule post-cleavage (DXd1-Linker in Fig. 1)^26^. The second compound corresponds to a neutralized version of the zwitterionic Lys-SMCC-DM1, in which the NH_3_^+^ and COO^-^ charged groups have been substituted with CH_3_ groups (SMCC-DM1 in Fig. 1).

#### pKa estimation procedure

**Fig. 4:**
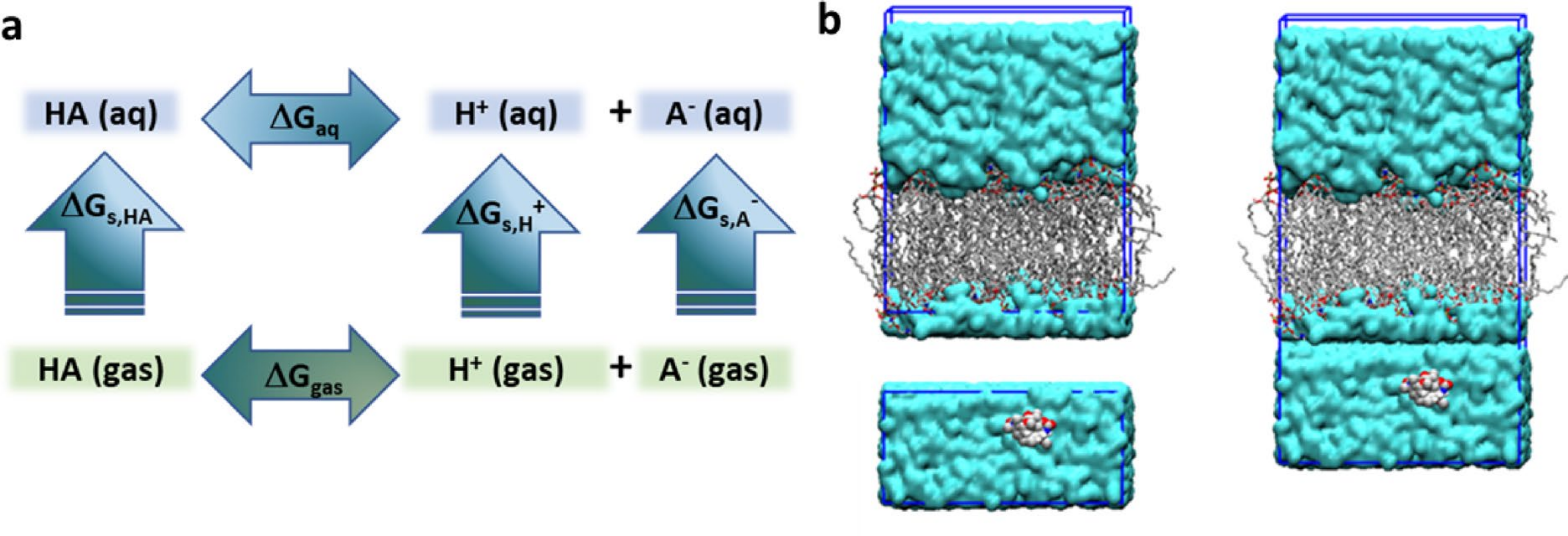
Methodology to obtain pKa and initial composition of simulated systems. (**a**) Born-Haber thermodynamic cycle for pKa calculation. HA corresponds to the protonated species*.* (**b**) On the left the POPC and solvated DXd1 boxes in the upper and lower positions are observed; on the right the composition used initially for the DXd1/POPC system is depicted.

Given the relevance of payload ionization, first-principles models have been applied to estimate their pKa data. Specifically, the Born-Haber thermodynamic cycle was chosen to estimate the pKa values for the ionizable molecules (see Fig. 4a)^41^. A mathematical expression to calculate the free energy ($$\Delta {G}_{aq}$$) of the equilibrium reaction: *HA* (aq) ⇌ $${A}^{-}$$ (aq) + *H*^+^ (aq) can be derived from the cycle in Fig. 3a: $$\Delta {G}_{aq}=$$$${G}_{gas}\left({A}^{-}\right)-$$$$6.28-{G}_{gas}\left(AH\right)+{E}_{aq}\left({A}^{-}\right)-{E}_{gas}\left({A}^{-}\right)-265.9-{E}_{aq}\left(HA\right)+{E}_{gas}\left(HA\right)+RT ln\left[24.46\right]$$, where G represents the sum of electronic and thermal free energies and E are electronic energies. All calculations were performed using the Gaussian 16 software^42^ using the M06-2X functional^43^. For conformational analysis after geometry optimization of the molecular structures, the 6-31G(d) and 6-31G + (d) basis sets were used for bases and acids, respectively, for frequency calculations on the optimized structures. The SMD implicit solvent model was used for the systems in aqueous solution^44^. The $$-6.28$$ and $$-265.9$$ values correspond to $${G}_{gas}\left({H}^{+}\right)$$ and $$\Delta {G}_{gas}\left({H}^{+}\right)$$, respectively, extracted from the literature^45^.

### In silico strategy for passive diffusion study

Conventional MD simulations face challenges in effectively capturing diffusion across the cell membrane, primarily because they often fail to reach the necessary timescales during simulations. Typically, conventional MD simulations are limited to short time scales, often in the nanosecond to microsecond range. As a result, they might not fully capture the entire diffusion process, especially for larger and complex molecules like cytotoxic payloads. To overcome this limitation, a simulation specialized strategy tailored to investigate this process has been implemented.

#### Force field

The GROMOS 53A7 united atom force field has been selected for all the components of the system^46,47^. The original force field was substantially enhanced in the ATB 3.0 implementation and validated against a broad range of experimental data, primarily enthalpies of solvation^48^. Although the mathematical forms used to represent interatomic interactions in the GROMOS 53A7, CHARMM36, and AMBER force fields appear similar and are typically calibrated using comparable experimental or computational data, the strategies guiding their optimization differ significantly. For instance, force fields like GROMOS have traditionally prioritized fitting parameters to reproduce thermodynamic properties—such as vaporization enthalpies, liquid densities, and solvation characteristics of small compounds. In contrast, force fields like AMBER and CHARMM tend to focus on accurately replicating molecular structural features.

Furthermore, the parameter development methods vary across force fields. CHARMM and AMBER employ detailed schemes that assign distinct bonded and non-bonded parameters based on each atom’s chemical environment, resulting in a large number of highly specific parameters. Meanwhile, GROMOS adopts a more generalized approach by treating molecules as assemblies of functional groups, applying a more limited parameter set. The former approach aims to capture fine-grained molecular details, while the latter emphasizes generalizability and reduces the risk of overfitting.

For small organic molecules commonly used in medicinal chemistry, particular emphasis is placed on accurately reproducing the free energy of solvation—the energy change associated with transferring a molecule from the gas phase into a solvent such as water. This property is especially valuable because it can be experimentally measured for a wide range of compounds and serves as a reliable benchmark for evaluating solute–solvent interactions. These interactions are crucial in biological contexts, such as ligand binding to proteins or compound partitioning into biological membranes, both of which involve transitions from aqueous solvation to more hydrophobic, less polar environments.

#### Structure of the lipid bilayer system

The lipid bilayer structure employed corresponds to that described by Poger et al.^49^, composed of 64 Palmitoyl Oleoyl Phosphatidyl Choline (POPC) lipids per monolayer. The structure was obtained from the Automated Topology Builder (ATB) website (https://atb.uq.edu.au). It uses a united atom representation of the lipid molecule, which accurately reproduces various experimental structural data of the bilayer in the biologically relevant liquid-crystalline phase (see Fig. 2b).

#### Constructing the simulation systems

The POPC bilayer structure was contained within a box with dimensions of [6.5, 6.5, 8.1] nm. As previously mentioned, this bilayer was equilibrated according to the specifications outlined by Poger et al.^49^. Each payload was placed in a separated water box of dimensions [6.5, 6.5, 5.0] nm using the tools provided in the GROMACS v.2023.2 software suite (http://www.gromacs.org)^50^. The solvated payload was subjected to a minimization protocol and a 10 ns equilibration step through MD simulation, during which all solute atoms were restrained to facilitate the relaxation of the water molecules surrounding the payload. To maintain the box dimensions, the simulations were performed in the NVT ensemble at a temperature of 310 K. Once equilibrated, the simulation box was combined with the POPC bilayer, as shown in Fig. 4b.

#### Simulation protocol

After system construction, the MD protocol involved the following steps:

- Minimization of the entire system to eliminate possible atomic bumps, mainly among water molecules coming from the lipid and payload original boxes. The Steepest Descent algorithm was used until maximum force converged below 1,000 kJ mol^-1^ nm^-1^.
- Equilibration of the minimized system with lipids and payload molecules restrained for 50 ns in the NPT ensemble to allow further relaxation of water molecules around the solutes.
- MD simulation of approximately 2 μs in the NPT ensemble until a steady-state was observed, characterized by payload adsorption at the interface between the polar head groups and the hydrocarbon core of the lipid bilayer.
- Production MD simulation of 1 μs in the NPT ensemble to collect and analyze structural data for each system.

Production MD simulations (step d) were performed in triplicate to assess the reproducibility of the observed structural properties. A time step of 2 fs has been selected, adopting the Single Point Charged model (SPC) for water molecules^51^ . Simulations were performed in two different ensembles, semi-isotropic NPT and NVT. The V-rescale thermostat with a time constant for coupling of 10 ps and a target temperature of 310 K for the equilibration and production stages in semi-isotropic NPT^52,53^ . In the case of semi-isotropic pressure coupling, the C-rescale scheme was followed by coupling separately the bilayer plane dimensions (X and Y) and the normal plane to the bilayer (Z dimension)^54^. Pressure, constant coupling and compressibility took the values of 1 bar, 5 ps, 4.5 × 10^–5^ bar^−1^, respectively. The Verlet buffer cutoff scheme implemented in GROMACS was used to calculate the non-bonded interactions using a van der Waals cutoff of 1.2 nm. For the estimation of electrostatic interactions, the PME method was applied with a cutoff value of 1 nm, an interpolation order of 4 and a Fourier spacing of 0.12^55^. Periodic boundary conditions were applied at all three directions to remove surface effects and mimic the bulk state.

#### Potential of mean force (PMF) calculation

A combination of non-equilibrium pulling and umbrella sampling was used to evaluate the PMF of each payload. The process was initiated from the final structures obtained in the previous unrestricted MD simulations and performed the following steps:

- Non-equilibrium payload pulling from the MD equilibrium configuration towards the bilayer interior was conducted. This involved using two reaction coordinates: the distance between the center of mass (COM) of the lower layer of P lipid atoms and the payload COM, and the center of mass of the charged group relative to the cylinder of phosphorus atoms in the lower lipid layer. Position restraints were imposed on the phosphorus atoms to prevent lipid molecule dragging. The designed protocol applied an umbrella potential with a force constant of K = 1,000 kJ mol^−1^ nm^−1^ to each coordinate, with a pulling velocity of 1 × 10^–5^ nm ps^−1^ over a total of 300 ns pulling simulation.
- Non-equilibrium pulling from the MD equilibrium configuration of the payloads towards the nearby water solution phase was conducted using the same two coordinates. In this case, position restraints for the phosphorus atoms were not considered.
- An umbrella sampling procedure was followed for each of those non-equilibrium MDs. From each pulling simulation, 72 frames equally spaced along the trajectory were extracted, corresponding to roughly a 0.4 Å separation between samples. Each frame underwent a simulation protocol similar to that described in the pulling steps, with coordinate velocities set to 0.0 nm ps^−1^ over a total of 30 ns time for each sample.
- The PMF was estimated using the Weighted Histogram Analysis Method (WHAM)^56^. The trajectories collected in the umbrella sampling procedure were subjected to WHAM analysis, considering information from both reaction coordinates. The last 20 ns of each window in the WHAM procedure were used, yielding the desired PMF and histograms of coordinate distribution along the total number of windows. Histogram distributions sufficiently overlapped in each coordinate were ensured.

## Supplementary Information

Supplementary Video 1.

## Data Availability

The atomistic structures of both DXd1 and DXd2 analogues were obtained from the Pubchem repository (PubChem CIDs: 118305111 and118305229). The atomistic structures of MMAE and MMAF were obtained from the PubChem repository (PubChem CIDs: 11542188 and 10395173). The three-dimensional structures of the maytansinoids DM1 and Lys-SMCC-DM1 were derived from the structural data in complex with tubulin present in the Protein Data Bank (PDB) database (PDB Entry: 5SBA). All data supporting the findings in this study are available within the paper, the Supplementary Video, or from the corresponding author upon reasonable request.
